# Patient Portals and Patient Engagement: A State of the Science Review

**DOI:** 10.2196/jmir.4255

**Published:** 2015-06-23

**Authors:** Taya Irizarry, Annette DeVito Dabbs, Christine R Curran

**Affiliations:** ^1^ School of Nursing University of Pittsburgh Pittsburgh, PA United States

**Keywords:** electronic personal health record, patient portal, patient engagement, meaningful use

## Abstract

**Background:**

Patient portals (ie, electronic personal health records tethered to institutional electronic health records) are recognized as a promising mechanism to support greater patient engagement, yet questions remain about how health care leaders, policy makers, and designers can encourage adoption of patient portals and what factors might contribute to sustained utilization.

**Objective:**

The purposes of this state of the science review are to (1) present the definition, background, and how current literature addresses the encouragement and support of patient engagement through the patient portal, and (2) provide a summary of future directions for patient portal research and development to meaningfully impact patient engagement.

**Methods:**

We reviewed literature from 2006 through 2014 in PubMed, Ovid Medline, and PsycInfo using the search terms “patient portal” OR “personal health record” OR “electronic personal health record”. Final inclusion criterion dictated that studies report on the patient experience and/or ways that patients may be supported to make competent health care decisions and act on those decisions using patient portal functionality.

**Results:**

We found 120 studies that met the inclusion criteria. Based on the research questions, explicit and implicit aims of the studies, and related measures addressed, the studies were grouped into five major topics (patient adoption, provider endorsement, health literacy, usability, and utility). We discuss the findings and conclusions of studies that address the five topical areas.

**Conclusions:**

Current research has demonstrated that patients’ interest and ability to use patient portals is strongly influenced by personal factors such age, ethnicity, education level, health literacy, health status, and role as a caregiver. Health care delivery factors, mainly provider endorsement and patient portal usability also contribute to patient’s ability to engage through and with the patient portal. Future directions of research should focus on identifying specific populations and contextual considerations that would benefit most from a greater degree of patient engagement through a patient portal. Ultimately, adoption by patients and endorsement by providers will come when existing patient portal features align with patients’ and providers’ information needs and functionality.

## Introduction

### Patient Engagement and Patient Portals

Patient engagement has been identified as an essential dimension of the multifaceted solution to the cost/quality crisis in US health care. The patient-centric definition of patient engagement by the Agency for Healthcare Research and Quality (AHRQ) is “the involvement in their own care by individuals (and others they designate to engage on their behalf), with the goal that they make competent, well-informed decisions about their health and health care and take action to support those decisions” [1]. AHRQ also defines patient engagement from a systems perspective as “a set of behaviors by patients, family members, and health professionals and a set of organizational policies and procedures that foster both the inclusion of patients and family members as active members of the health care team and collaborative partnerships with providers and provider organizations” [1].

Currently, there is an increasing awareness of health care system’s responsibility to provide easily accessible ways for patients to be engaged in their own care by creating effective partnerships that lead to the patient’s ability to make competent and well-informed decisions [2]. While an electronic personal health record (ePHR) tethered to an electronic health record (EHR), also known as a patient portal, is currently recognized as a promising mechanism to support greater patient engagement, questions remain about how health care leaders, policy makers, and designers can encourage adoption by both providers and patients and what factors might contribute to sustained utilization.

### Definition and Background of Patient Portals

An ePHR that directly links, or is “tethered”, to an EHR is most commonly referred to as a patient portal. In general, patient information from the EHR such as the problem list, allergies, and lab test results populate the patient portal. In some instances, patients may enter data to populate the EHR. In contrast, an untethered ePHR is under the control of the patient. This means an individual manually enters all information or grants permission for the information to be transferred to the ePHR, from a specific source like a laboratory or pharmacy, and determines who will have access. Thus, the value of an untethered ePHR is determined by a person’s willingness to manage and maintain their ePHR information. Because there is little that health care organizations can do to initiate patient engagement using an untethered ePHR, this literature review is focused exclusively on the patient portal, directly linked to an EHR.

Patient portals were introduced and adopted by a few large health care organizations in the late 1990s (eg, MyChart at the Palo Alto Medical Foundation and Indivo at Boston Children’s Hospital) [3,4]. However, patient portals did not gain widespread use until 2006 when several initiatives coincided, including the launch of ePHRs by Microsoft and Google, the awarding of Centers for Medicare and Medicaid Services (CMS) contracts to private firms to conduct feasibility studies of ePHRs using existing claims data from Medicare programs, and Blue Cross and Blue Shield Association and America’s Health Insurance Plans’ announcement to develop data-sharing programs that would ultimately support ePHR development [5]. These initiatives also coincided with the broad social movement towards adoption and daily use of powerful information and communication sharing tools such as smartphones and social media, illustrating the readiness of the general population to embrace technology in a new socially interactive way.

The current principal driver of patient portal development is the meaningful use (MU) criteria of the CMS EHR incentive program [6]. Features mandated by MU that directly relate to patient portal functionality include providing (1) a clinical summary to the patient after each visit, (2) secure messaging (SM) between patient and provider, (3) ability to view, download, and transmit personal health record data, (4) patient specific education, (5) patient reminders for preventative services, and (6) medication reconciliation [7]. While these criteria clearly outline tasks and goals, they do little to reflect the value proposition to the end users (patients and providers) or the steps required to engage patients in a sustained and relevant way. Therefore, an aim of this review was to explore the current research addressing the encouragement and support of patient engagement through the patient portal.

## Methods

### Search Strategy

Due to the advances in technology and consumer readiness in the mid-2000s, the review was limited to recent literature to better reflect current trends in design, functionality, and perceived user readiness of patient portals. We reviewed literature from 2006 through 2014 in PubMed, Ovid Medline, and PsycInfo using the search terms “patient portal” OR “personal health record” OR “electronic personal health record”. Bibliographies and the literature reviews from these sources were used to identify additional studies [8,9]. Initial inclusion criteria were (1) original, peer-reviewed, qualitative, and quantitative research of tethered ePHRs or patient portals, (2) English language, and (3) available in full text. The final inclusion criterion was that the studies reported on the patient experience and/or ways that patients may be supported to make competent health care decisions and act on those decisions using patient portal functionality. Studies were not targeted to any particular patient subgroup, disease, or clinical setting.

**Figure 1:**
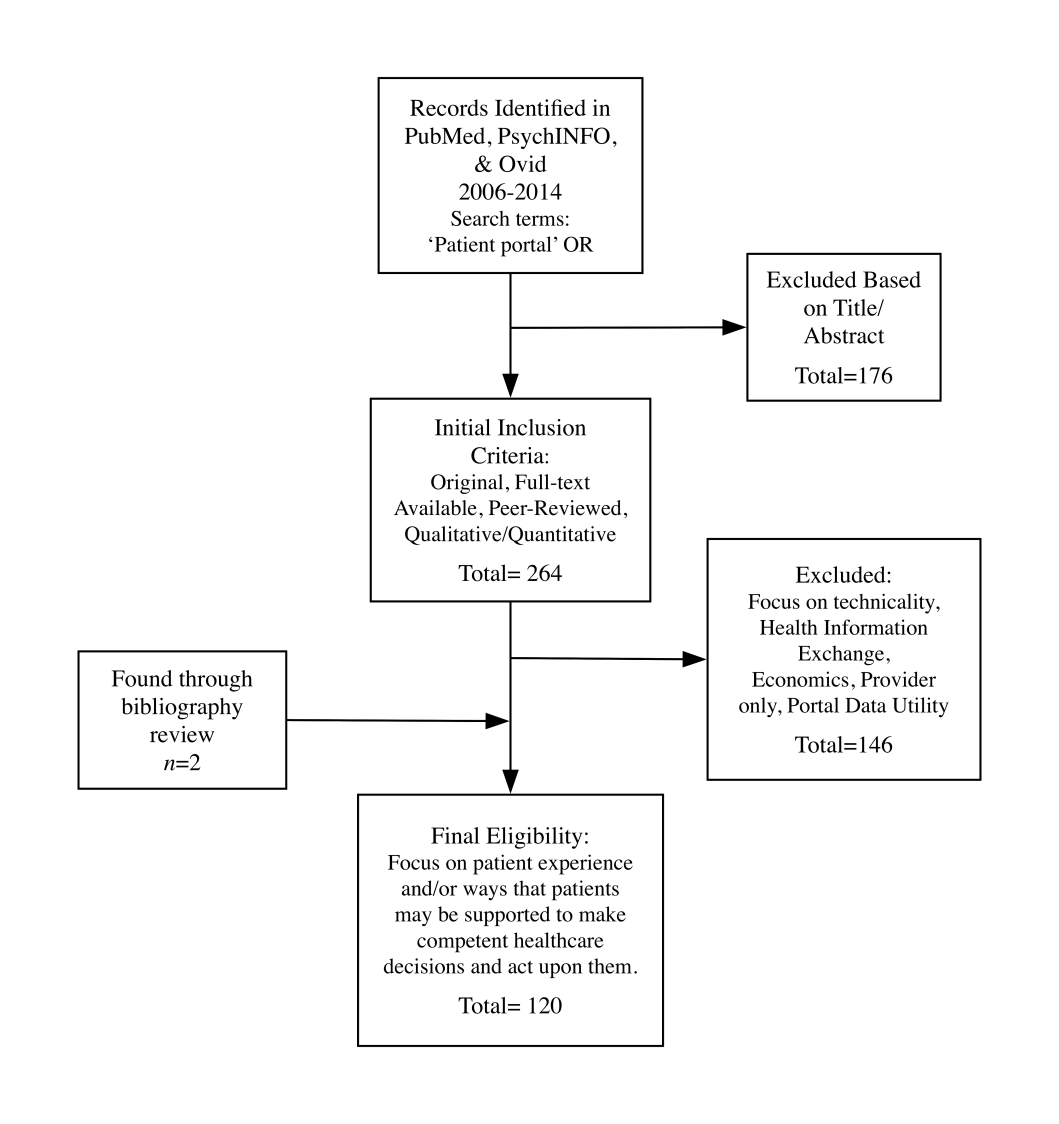
Literature review flow chart.

Of the 440 articles identified by the search, 176 were excluded based on title and abstract. Further review based on the final inclusion criterion resulted in 120 articles, which were reviewed in depth (see Multimedia Appendix 1 for summaries of each). Excluded articles focused on the provider perspective only, technicalities of patient portal implementation (eg, policy issues, safety, security), implications for Health Information Exchange, economics impacts, or the utility of patient portal data for research purposes (see Figure 1).

## Results

### Overview

We grouped the studies into five major topics based on the research questions, explicit and implicit aims of the studies, and related measures addressed. The topics identified included patient adoption, provider endorsement, health literacy, usability, and utility (Table 1). Of the 120 articles that were reviewed, 66 (55.0%) were non-experimental descriptive, 26 (21.7%) were qualitative or mixed-methods, 14 (11.7%) were randomized controlled trials, 10 were pilot studies or case reports (8.3%), and 4 were cohort studies (3.3%) (Table 2). Only 11 articles explicitly identified a guiding theoretical framework, with the Chronic Care Model being the most common among them. The year 2011 was a turning point in the number of published articles, which coincides with the initiation of CMS EHR incentives program. The topical areas that showed the greatest increase in volume were patient adoption and utility (Table 1). See Multimedia Appendix 1 for a brief description of each article and the topical areas addressed. The following section describes each topical area and discusses relevant implications for research, development, and implementation of patient portals.

**Table 1:** Summary of articles on categories of patient portals for patient engagement.

Year	Provider endorsement	Health literacy	Usability	Patient adoption	Utility	Total # of articles
2006	0	0	0	1	2	3
2007	1	1	1	2	3	4
2008	0	1	1	4	6	8
2009	0	0	1	3	4	7
2010	1	2	2	7	7	11
2011	3	3	2	11	8	17
2012	1	3	3	11	10	16
2013	2	3	5	12	17	27
2014	0	3	5	11	19	27
Total	8	16	20	62	76	120

**Table 2:** Levels of evidence adapted from Melnyk & Fineout-Overholt, 2005*.*

Type of study		Level of evidence	# of studies
RCT		2	14
Cohort/Quasi-experimental		3	4
**Descriptive**		4	
	Non-experimental (survey, correlational, etc)		66
	Qualitative/Mixed method		26
Pilot study/case report		5	10

### Patient Adoption

Before a patient portal can serve as a tool for individuals to become more engaged and involved in their own care, patients must first adopt it. CMS 2014 stage 2 MU regulations define adoption in terms of institutional reporting for reimbursement and require that 5% of the institutions’ patient population (1) download or view electronic health information and (2) use secure electronic messages (eg, email) [6]. However, in our review, various operational definitions of adoption were used. For example, many observational studies used usage data of the initial login to the patient portal site to represent adoption; others used data from surveys about patients’ intention to use the portal. Several randomized controlled trials (RCTs) used rates of patient portal intervention adherence to study protocol to define adoption, and for some of these trials, those who completed the studies were considered adopters; in others, adoption was defined as the frequency of intervention use.

Of the 62 articles [5,10-70] that focused on or described patient portal adoption as part of the report, six RCTs included detailed descriptions of intervention group participants who completed the study (and therefore were considered adopters) in comparison to those who did not. We found 12 qualitative or mixed-method studies that collected data about adoption from patients through focus groups or semistructured interviews; 21 studies focused on interest and barriers to adoption for specific populations or patient portal functions (eg, elderly, safety-net, human immunodeficiency populations, secure messaging, prescription refills).

The term “digital divide” is often used to describe major potential barriers to access of electronic tools such as a patient portal and refers to disparities among subgroups based on access to the Internet and computer literacy. However, this term does not encompass the many other factors that may contribute to adoption such as language barriers, age, race and ethnicity, social economic status, and level of patient activation [32,50,54,71]. Several studies examining adoption have shown that ethnic minorities (African American, Latino, Asian) and patients who are younger (under 35 years), healthier, and less educated were less likely to adopt patient portals [15,55,72]; however, results are mixed regarding gender differences [50,63]. People with disabilities and chronic conditions, frequent users of health care services, and caregivers of elderly parents or children tend to have the most interest in patient portals [28,50,62,73]. Other important factors of patient portal adoption include provider acceptance and promotion, and usability of the patient portal interface including ease of registration, navigation, and perceived privacy and security [18-20,74].

### Provider Endorsement

Provider endorsement and continued engagement with the patient portal have been identified as important factors in a patient’s decision to adopt and continue to use the patient portal functions to achieve and sustain anticipated positive outcomes [19,75]. Of the 8 articles that addressed physician endorsement [12,19,34,76-80], 5 studies were qualitative or mixed-method studies, and one RCT included a retrospective survey of physicians’ use and satisfaction.

Four of the studies sought to capture attitudes of clinicians towards patient portals prior to having firsthand experience interacting with them. Prior to actual use of patient portals, clinicians expressed concerns related to patient engagement including: the potential for inducing patient anxiety regarding test results; the accuracy of patient entered data; the potential liability for tracking and acting on critical clinical information, such as blood glucose levels and blood pressure readings; implications for changes in the patient-provider relationship; and the anticipated increased workload [34,77,78,81].

Retrospective studies showed that the pre-portal concerns regarding patient anxiety about test results were not justified as demonstrated by numerous patients who found the test result feature one of the most useful [82]. In addition, while perceived increases in workload and duration of clinic visits varied among studies, clinicians believed patients were more interested in participating in their care and found that verifying the additional information in the patient portal provided during face-to-face visits was helpful, thus eliminating the accuracy concern [19]. Overall, the workflow of individual providers and the health care team as a whole, including nurses, pharmacists, support staff, and physicians, must be adapted in order to incorporate patient portal functionality, and the patient engagement it allows, into the delivery of preventative services and illness management processes [45].

### Health Literacy

The definition of health literacy developed for the National Library of Medicine and used by the Healthy People 2010 initiative is “the degree to which individuals have the capacity to obtain, process and understand basic health information and services needed to make appropriate health decisions” [83]. Of the 16 studies that specifically addressed health literacy [11,14,30,40,42,64,65,74,84-91], the majority included self-reported health literacy measures via survey questions or open-ended questions; only Noblin et al (42) and Taha et al [91] included validated health literacy measures. Four studies [64,85,88,91] identified conceptual knowledge, numeracy, and computer skills as particularly important literacy factors that contributed to successful patient engagement via a patient portal.

Noblin et al [42] found that 65% of participants who intended to adopt the outpatient clinic’s patient portal had a higher eHealth literacy score than those who were not interested in patient portal adoption. Taha et al [91] results indicated that if health texts involved numeric concepts, users encountered problems, even if they were considered to have “adequate” health literacy. These studies underscore the importance of evaluating health literacy and health numeracy separately in order to identify specific risk factors and design flaws that could impact patient comprehension and ultimately jeopardize the accuracy of patient input and interpretation of results.

Four studies directly addressed the impact of health literacy of intended users on the successful completion of specific tasks [64,84,88,92]. Results showed that patients responded better when medical jargon and abbreviations were translated into “patient friendly” language. These results echo Haggstrom et al [85] and Monkman & Kushniruk’s [88] findings of the dangers of low health and computer literacy to safe and effective use of patient portals.

Schnipper et al [92] and Sox et al [84] revealed that, despite patient involvement in early design and testing of patient portals, subsequent scenario-based usability testing uncovered navigation difficulties primarily due to the unfamiliarity with complex medical language and confusion of how and when to correct identified errors. Monkman & Kushniruk [88] suggest that including health literacy assessments in usability testing of consumer health information systems, such as patient portals, would inform the design of systems for better navigation, data input, and conceptual understanding of health information included throughout the patient portal.

Monkman & Kushniruk [88] also proposed a specific heuristic for health literacy whose purpose is to identify and categorize when clinical information within the patient portal would most likely be misunderstood by a layperson who does not possess a health care background. This study, along with several other qualitative studies showed that specific health topics (eg, medications, lab results, and allergies) required extra attention to designing with health literacy considerations in mind [45,89,93]. Proposed navigation and aiding tools that increased patients’ ability to understand their personal health information more fully include integrating links to definitions of terms and detailed explanations, using movies and illustrations, substituting lay language for medical terminology and using graphs to track trending data, such as blood pressure and blood glucose levels [84,85,94].

### Usability

Usability testing is the term used to describe the assessment of how easy a user interface is to operate. The word “usability” also refers to methods for improving ease of use during the design process [95]. One such method is heuristic evaluation, a method of testing a preliminary prototype by examining the interface and judging its compliance with recognized usability principles (ie, “heuristics”). Further iterative usability testing is accomplished using a series of prototypes and participatory scenario-based and “think-aloud” sessions with intended users in order to redesign the interface and workflows to better match user needs and preferences. Early usability testing, and its role in patient portal design, is important because it directly impacts whether or not a patient can easily adopt a patient portal. It also impacts the ability of the user to successfully navigate portal functions, accurately input information, and comprehend the information presented, ultimately contributing to its usefulness as a tool for patient engagement.

Of the 20 studies that addressed usability of patient portals, 6 performed some form of heuristic and usability testing with objective observation and various forms of “think aloud” sessions [25,84,85,92,94,96]. Only Schnipper et al [92] included usability testing of both the clinician and patient interfaces. The remaining 14 studies assessed users’ subjective satisfaction and ease of use with questionnaires and/or interviews to evaluate overall adoption and utilization [11,38,45,47,48,64,65,73,82,88,89,91,97,98].

Schnipper et al [92] addressed the needs of both end users (ie, clinicians and patients) in the usability testing of a medication management module embedded within the patient portal. The study highlighted the need for end user-specific interfaces and functionality in order to make the user experience easier and more efficient, thus demonstrating its value and promoting sustained use. For patients, this meant striking a balance between free-text, structured, and coded data fields in order to leverage the usefulness of patient-entered data without confusing or overwhelming patients. For example, drop-down menus and scrolls bars were found to be less confusing and more efficient than dynamic text boxes that would react to the word being typed when inputting data, such as medications and allergies. In the case of clinicians, this meant integrating the clinician side of the application with their workflow so that clinicians could verify and correct patient-entered data while simultaneously facilitating the flow of that data into the EHR.

Much of the literature surrounding usability confirms that adoption and sustained use of technology are directly related to ease of navigation and the perceived usefulness of the available information [99]. While nearly all the patient portal usability studies that used subjective assessments showed positive results for ease of use and satisfaction, the in-depth objective usability studies were more effective at uncovering a variety of barriers to safe and effective use.

### Utility

Utility refers to the availability of needed features. Utility and usability are equally important and together determine whether something is useful [99]; 76 studies focused in some way on patient portal utility [5,12,13,15,19,22,23,25-27,30,34,37,41,44, 47,52,53,56,57,59,60,64,65,69,70,79,82,84-87,89,90,92,96,98,100-137]. The majority of descriptive, qualitative, and mixed-method studies focused on eliciting patient preferences for specific functions. Patients preferred functions that offered convenience, such as an easy way to contact and communicate with providers, order prescription refills, and access multiple family medical records. Easy-to-read, printer-friendly summaries were also viewed as helpful for sharing information with family members and providers who did not have patient portal access. The top two patient portal qualities that were deemed most utilitarian for patients were personalization and collaborative communication between patients and providers [67,138].

### Personalization

While numerous descriptive and qualitative studies attest to the desire for personalized patient portal functionality, there is little research about what kind of personalization would lead to greater patient engagement. Currently, the greatest research focus is on chronic disease medication management and preventative services. Only 3 RCTs specifically tested the efficacy of patient-tailored interventions [13,30,90]. Grant et al [13] provided patient-tailored decision support and enabled the patient to author a “Diabetes Care Plan” for electronic submission to the physician prior to upcoming appointments. This intervention led to increases in pre-visit use of the patient portal and increased rates of diabetes-related medication adjustment at 12 months. Krist et al [62] provided a personally tailored list of prevention recommendations and found that at 16 months, 1 in 4 users were up-to-date on all preventive services—nearly double that of non-users. Sequist et al [30] sent personalized electronic messages that included (1) alerts for overdue health screenings and information on screening options, (2) a mechanism for patients to submit requests to schedule screening examinations, and (3) a link to a Web-based tool for patients to assess their personal risk of colorectal cancer. Findings showed that screening rates were significantly higher at 1 month for patients who received electronic messages than for those who did not, but the difference was no longer significant at 4 months.

### Collaborative Communication

Collaborative communication refers to the ability for patients and providers to share timely and pertinent information, enabling patients to participate as active members of the care team beyond the hospital or clinic setting. SM and medication reconciliation are the two most common patient portal functions that offer the opportunity for such communication. Both functions also pose the greatest potential changes to provider workflow and overall impact on the patient-provider relationship.

For example, the difficulty aligning information management tools with current provider workflow and care delivery priorities was highlighted in a study of an interactive medication reconciliation module that emailed primary care physicians when a patient added or changed information [106]. Results showed that patients were willing and able to annotate their medication list, offering the most up-to-date and complete information, but email notifications were ineffective at prompting providers to update the EHR medication list outside of a clinic visit [106]. Thus, while the notion of designing patient portals to support patient involvement in their care, such as opportunities for their participation in medication reconciliation, shows promise, their effectiveness will depend on the ability to better incorporate these functions into provider workflow and delivery of care.

Other implications of electronic forms of communication via a patient portal are the potential to improve efficiency by way of substituting SM for face-to-face encounters and using SM reminders to decrease missed appointments and promote timely preventative care. However, research shows mixed results leading researchers to believe that the relationship between SM and utilization is more complex than the simple substitution of online for in-person care suggests. For example, while an earlier study at Kaiser Permanente showed a decrease in face-to-face encounters after the initiation of SM [22], a subsequent study in a different Kaiser region showed the opposite effect [115]. A study done at the Mayo Clinic, aimed at clarifying this discrepancy, focused on frequency of messages, long-term use, and importance of SM among certain subgroups [121], which showed neither an increase nor decrease in face-to-face provider visits with the use of SM.

SM is also being used as a one-way communication tool to deliver reminders for preventative care and appointments. A 2011 study at seven Duke medical clinics showed that email reminders, in combination with scheduling functionality within the patient portal, demonstrated significant declines in “no-shows” [27]. A meta-analysis and systematic review by Guy et al [139] demonstrated a substantial increase in the likelihood of attending clinic appointments when patients received SM reminders. Perhaps the most encouraging results with SM were the large reduction in missed appointments among historically disadvantaged groups, such as Medicaid recipients, the uninsured, and black patients [27].

SM reminders via email have also been shown to be generally successful at encouraging higher rates of preventative services use. For example, a multi-practice randomized controlled trial showed improvement in the rates of certain preventive screenings and vaccinations, but preventative services as a whole were not impacted [113]. Findings suggest that SM reminders are most effective when they are tailored to the population and context, thus targeting specific goals such as herpes zoster vaccinations for older adults, or pediatric preventative care visit reminders for parents [119,129].

## Discussion

### Principal Findings

The current principal driver of patient portal development is CMS and Medicaid EHR incentive program meaningful use (MU) criteria [6]. While MU criteria clearly outline requirements of basic functionality and targeted adoption rates, they do not delineate the steps or features required to engage patients in a sustained and relevant way. Presently there is no clear definition of patient portal adoption beyond the minimum use requirements outlined in the MU criteria. However, in order for health care institutions to track the success of patient portals in terms of patient engagement, a multi-dimensional definition of portal adoption should include both motivating factors for initiation and use over time A definition of this kind would inform a set of universal quality and efficiency reporting measures beyond the current minimal MU criteria to include more relevant patient engagement data.

Current research has demonstrated that patients’ interest and ability to use patient portals is strongly influenced by personal factors such age, ethnicity, education level, health literacy, health status, and role as a caregiver. Health care delivery factors, mainly provider endorsement and patient portal usability, also contribute to patients’ ability to engage through and with the patient portal.

While health literacy has been identified as an important factor in the successful use of patient portals, few studies have used validated health literacy measures, making it difficult for future research to build on the findings. Research demonstrates that specific aspects of health literacy, mainly numeracy and familiarity with medical terminology, greatly impact the ability of patients to accurately input data and interpret the information provided in the patient portal. The direct relationship between health literacy and effective use of the patient portal supports the argument for the use of specific health literacy heuristics as part of overall usability testing.

Research also demonstrates that objective testing (as opposed to solely subjective) should also be a part of patient portal usability testing. Although objective usability testing is expensive and time consuming, studies demonstrate the need for continued work in this area in order to ensure patient portal interfaces promote patient comprehension and data entry accuracy. The promotion of content accuracy and patient comprehension impacts the overall usefulness of the information for both patients and providers.

The perceived usefulness of patient portals from the providers’ perspectives cannot be underestimated. Provider endorsement is one of the most influential factors impacting patients’ initial adoption, as well as its continued use as a tool for collaborative communication [20]. Yet, current research demonstrates the difficulty in aligning information management tools, such as the patient portal, with current provider workflow and care delivery priorities.

While current development and research is focused on demonstrating feasibility and efficiency of medication reconciliation and SM reminders, the research has revealed roadblocks to successful implementation rooted in the lack of provider workflow adaptations A greater understanding of the essential adjustments in provider workflow, including potential changes in the roles and responsibilities of the care team overall, is necessary in order to translate findings into practice. Few studies have focused on exploring how patient portal use should unfold within the context of the patient-provider interaction, or how it might impact the overall organization and workflow of the health care team including potential liability concerns, reimbursement, and relationships with patients.

Ultimately, successful implementation requires health care institutions to invest time and resources to systematically assess the health needs of their specific patient and caregiver populations, their individual stages of readiness to adopt a patient portal, and the types of assistance needed to do so [140]. Ideally, interactive sites would collect information on individuals’ health, health behaviors and personal goals, and assess health literacy and functional ability, which would then inform the adaptation of the patient portal to accommodate the needs of the individual and/or what additional or alternative resources may be useful [2]. Such adaptations include personalized content and tailored data presentations specifically designed to enhance interpretation and comprehension of key personal health concerns and timely and pertinent action steps.

In addition, external environmental and contextual factors, such as distance between patient and clinic, and complexity and trajectory of health concerns, may impact which form of access is preferred for a specific person, provider, location, and situation. Future directions of research should focus on identifying specific populations and contextual considerations that would benefit most from a greater degree of patient engagement through a patient portal. This information could then lead to the creation of health care service policies that promote the use of a patient portal by both providers and patients within the most appropriate settings.

### Conclusions

If institutions are to engage patients via the patient portal in a way that encourages them to become active members of the care team, support their competence in making health-related decisions, and help them to act on those decisions, institutional leaders must consider the contributing factors that impact efficacy and sustained use of patient portals. According to this review, these factors include attention to the topical areas of patient adoption, provider endorsement, health literacy, usability, and utility. Ultimately, adoption by patients and endorsement by providers will come when existing patient portal features align with patients’ and providers’ information needs and functionality. Conceptualizing patient portals as a dynamic component of the patient-provider relationship and health care delivery system as a synergetic whole, rather than an isolated repository of information or a set of disconnected functions meant to collect patient data for provider use, may help to inform future research, improve patient portal design, and efforts to promote adoption and effectiveness.

## Appendix

Multimedia Appendix 1 A brief description of each article and the topical areas it addresses.

## Abbreviations

AHRQ: Agency for Health care Research and Quality
ePHR: electronic personal health record
EHR: electronic health record
CMS: Centers for Medicare and Medicaid Services
MU: meaningful use
SM: secure messaging
