# Supplementary material for: Patient Portals and Patient Engagement: A State of the Science Review
Source: J Med Internet Res. 2015 Jun 23;17(6):e148. doi: 10.2196/jmir.4255 (PMC4526960; doi:10.2196/jmir.4255)
Supplement: Multimedia Appendix 1 [file jmir_v17i6e148_app1.pdf]

Year	Author (ref #)	Study Design	**Patient Engagement** (P.A.= Patient Adoption, P.E.= Patient Endorsement, Us. = Usability, Ut.= Utility H.L.= Health Literacy					Major Findings
			P.A.	P.E.	Us.	Ut.	H.L.	
2006	Grant [101]	Case report				1		Description of a portal that enables patients to author a "Diabetes Care Plan" with patient-tailored decision support. Additional physician view of the patient's Diabetes Care Plan designed to fit into typical EHR clinical workflow.
2006	Tuil [100]	Descriptive				1		Patients rated most functions as useful; preferred personalized to general functions; some functions require further development.
2006	Weingart [10]	Descriptive	1					New enrollees logged in most frequently in the first month; most often examined laboratory and radiology results and sent clinical messages to their providers. Enrollees were younger and more affluent and had fewer medical problems than non-enrollees.
2007	Hess [102]	Mixed-Method				1		Patients felt that the system would enhance communication with the office, and that the reminder system would be helpful; liked having access to laboratory tests remotely; frustrated when tests were not released and messages not answered.
2007	Keselman [11]	Mixed-method	1		1	1	1	Providers' notes, lab test results and radiology reports were the most difficult records sections for lay reviewers; medical terminology, lack of explanations of complex concepts (e.g. lab test ranges) was the most common comprehension barriers.
2007	Kim [103]	Descriptive				1		Some patients may not keep their Patient portal up-to-date because they don't value, can't access, or don't understand certain categories of their health information.
2007	Wald [12]	Case report	1	1		1		Lab test results released to patients resulted in positive patient and staff feedback; no noticeable rise in patient concerns/distress; half of patients accessed reference information linked to the result.
2008	Bergman [104]	Qualitative				1		Significant themes: 1) issues about teens' control of their own healthcare 2) enthusiasm about the use of a portal to access providers, seek health information, and make appointments 3) concerns about confidentiality.
2008	Grant [13]	RCT	1			1		Pre-visit use of patient portal increased rates of diabetes-related medication adjustment. Low rates of portal registration and good baseline control among participants limited the intervention's impact on overall risk factor control.
2008	Green [105]	RCT				1		Pharmacist care management delivered through the portal improved BP control in patients with hypertension verses usual care or portal access only.
2008	Greenhalgh [14]	Qualitative	1				1	Key factors influencing portal adoption: 1) the nature of illness (especially if likely to lead to emergency care needs); experience of healthcare and government surveillance; level of health literacy; and trust and confidence in the primary healthcare team. Common misconceptions: confusion about what data it contained and who would have access to it.
2008	Lafky [15]	Mix-method	1			1		Users with disabilities differ from others in their portal preferences: particularly motivating factor for disabled individuals is the way a portal will function when emergency services are required.
2008	Schnipper [92]	Descriptive Usability testing			1	1		Usage and satisfaction data indicate that patients found the module easy to use, felt that it led to their providers having more accurate information about them and enabled them to feel more prepared for their forthcoming visits.
2008	Staroselsky [106]	Descriptive				1		Patients using a portal had just as many discrepancies between medication lists and self-report as those who did not, and notifying physicians of discrepancies via e-mail had no effect.

2008	Zickmund [16]	Qualitative	1					Interest in the portal was linked to dissatisfaction with provider responsiveness, the difficulty obtaining medical information, and logistical problems with the office. Disinterest in the portal was linked to satisfaction with the provider-patient relationship, difficulty in using the portal, and fear of losing relationships and e-mail contact with providers.
2009	Britto [94]	Descriptive Usability testing			1			Despite prior heuristic testing, scenario-based testing demonstrated difficulties in navigation, medical language complexity, error recovery, and provider-based organizational interface design.
2009	Chen [17]	Descriptive	1					Annual age/sex-adjusted total office-visit rate decreased 26.2% (n=225,000) at Kaiser Permanente Hawaii between 2004 and 2007. Scheduled telephone visits increased more than eightfold, and secure e-mail messaging, nearly six-fold between 2005 and 2007.
2009	Kim [109]	Mix-method				1		47% of patients used the portal only on a single day (n=77). Use highly correlated with the availability of in-person assistance; 77% of user activities occurred while the assistance was available. Residents' ability to use the portal was limited by poor computer and Internet skills, low health and computer literacy, and limited physical/cognitive abilities.
2009	Leveille [108]	RCT				1		85% of intervention group (n=121) and 80% (n=120) of the control reported discussing their screened condition during their PCP visit. More intervention than control patients reported their PCP gave them specific advice about their health (94% vs. 84%) and referred them to a specialist (51% vs. 28%). Results showed no differences in detection or management of screened conditions, symptom ratings, and quality of life between groups.
2009	Roblin [18]	Cohort study	1					Of 1,777, 34.7% registered with KP.org between Oct 2005 and Nov 2007. Among African Americans, 30.1% registered, compared with 41.7% of whites. In the hazards model, African Americans were again less likely to register than whites (hazard ratio [HR] = 0.652, 95% CI: 0.549-0.776) despite adjustment. Those with baseline Internet access and higher education levels were more likely to register.
2009	Wald [107]	Descriptive				1		Diabetes care plan took 5-9 minutes to be created by the patient. 60% felt more prepared for their visit and 53% felt it helped them give more accurate information to their provider (n=37).
2009	Weitzman [5]	Mixed-method	1			1		Perceived value for portal was highest around abilities to view, update, and share health information with providers. Patient preferences were highest for embedded searching, linking, and messaging capabilities; within-system tailored communications; and linkages between self-report and clinical data.
2010	Greenhalgh [25]	Mix-method	1		1	1		Patients perceived the portal as neither useful nor easy to use and its functionality aligned poorly with their expectations and self-management practices. Those who used email-style messaging were positive about its benefits, but enthusiasm apart from the three early adopter clinicians was low.
2010	Kahn [24]	Descriptive	1					Those who rated access to their patient portal as important were more likely to be Hispanic and Internet users and less likely to be age 65 and above or individuals whose doctors always ensured their understanding of their health.
2010	Nazi [23]	Descriptive	1			1		Direct feedback from early adopters of My HealtheVet portal demonstrated high satisfaction, highly likely to return to the site, and recommend the site to other veterans. Most veterans currently visited the site for pharmacy-related features.
2010	Reti [111]	Qualitative-Expert Opinion				1		Semi-structured interviews conducted in 7 large early portal adopter organizations. Most organizations enable data access for proxies and minors. No organization allowed patient views of progress notes, and turnaround times for reporting of normal laboratory results could be up to 7 days.
2010	Sarkar [74]	Descriptive					1	Those with limited health literacy had higher odds of never signing on to the patient portal compared with those who did not report any health literacy limitation (OR=1.7, 1.4 to 1.9). The relationship between health literacy and patient portal use endured among those with internet

								access, (OR=1.4, 95% CI 1.2 to 1.8).
2010	Sox [84]	Qualitative			1	1	1	Results informed the personalization of the ADHD application by (1) simplifying the visual environment, (2) supporting users with limited health literacy or technology experience, (3) populating the application's welcome screen with pictures of culturally diverse families.
2010	Wald [19]	RCT	1	1		1		Among 2,027 patients invited to complete a portal eJournal, 70.3% submitted one and 71.1% of submitters had one opened by their provider. Surveyed patients reported they felt more prepared for the visit and their provider had more accurate information about them.
2010	Wald [20]	Case report	1					Marketing limitations, leadership concerns, and limited staff engagement characterized the low-enrollment in some practices, but not the others. These factors, along with other characteristics such as location and patient demographics, should be explored in future research to identify best practices for successful adoption of a patient portal.
2010	Wen [21]	Descriptive	1					Approximately 86% of participants rated access to patient portal as important (n=7674). However, only 9% of them used the Internet for portal tracking. Those who rated electronic access to their portal as important were more likely to be Hispanic and Internet users and less likely to be age 65 and above or individuals whose doctors always ensured their understanding of their health.
2010	Wiljer [110]	Quasi-experimental				1		311 breast cancer patients completed demographic and pre-assessment questionnaires, 250 registered to use the online intervention, and 125 participants completed all required study elements. Participants generally found the portal easy to use; the perceived value of improved participation was not detected in self-efficacy scores. Having access to personal health information did not increase anxiety levels.
2010	Zhou [22]	Descriptive	1			1		Of 35,423 people with diabetes, hypertension, or both, the use of SM within a two-month period was associated with a statistically significant improvement in effectiveness of care as measured by the Healthcare Effectiveness Data and Information Set (HEDIS); the use of e-mail was associated with an improvement of 2.0-6.5 percentage points in performance on other HEDIS measures such as glycemic (HbA1c), cholesterol, and blood pressure screening and control.
2011	Ancker [26]	Descriptive	1			1		Patients with chronic conditions were more likely to become repeat users. The odds of repeat portal use increased with white race, English language, and private insurance or Medicaid compared to no insurance. Racial disparities were small but persisted in models that controlled for language, insurance, and health status.
2011	Do [96]	Pilot study			1	1		Key lessons learned related to data-transfer decisions (push vs. pull), purposeful delays in reporting sensitive information, understanding and mapping patient portal use and clinical workflow, and decisions on information patients may choose to share with their provider.
2011	Goel [28]	Descriptive	1					63% of respondents (n=159) not enrolling reported never attempting enrollment despite remembering receiving an order. 30% of those non-enrolling reported not attempting because of negative attitudes toward the portal and 8% reported computer related obstacles. Black respondents were less likely than white respondents to consider features assisting self-management such as getting test results as important. Adjusting for age, gender, education, and chronic disease did not substantially change results.
2011	Goel [29]	Descriptive	1					Overall, 69% of 7,088 patients enrolled in the patient portal. All minority patients were significantly less likely to enroll than whites: 55% blacks, 64% Latinos and 66% Asians compared with 74% whites. Disparities persisted in adjusted analyses, except for Asians. Younger patients were significantly less likely to solicit provider advice or request medication refills than any other age group in unadjusted and adjusted analyses. Similarly, male patients were less likely to solicit provider advice than women in all analyses.

2011	Haggstrom [85]	Descriptive			1	1	1	25% of users successfully completed registration (n=24). Users preferred prescription numbers to names, due to privacy concerns. Users wanted to print their information to share with their doctors, and questioned the value of MyHealtheVet search functions over existing online health information. Portal registration must balance simplicity and security; healthcare organizations should build trust for portal health content.
2011	Horvath [27]	Descriptive	1			1		15.7% of portal enrollees across seven clinics (n=58,942), scheduled 198,199 appointments with an overall no-show rate of 9.9%. Large reductions in no-show rates were seen among historically disadvantaged groups: Medicaid holders, uninsured patients, and black patients.
2011	Jung [36]	Descriptive	1					Results from portal consumer eVisits, in aggregate and in 4 distinct ambulatory practices indicate that out of 10,532 portal users, the 336 patients who submitted 446 eVisits between April 1, 2009 and May 31, 2010 are younger, predominantly female, not retired, and in poorer health condition on average. Practice indicator was a significant predictor of eVisit usage- researchers speculate this may be due to varying strategies used to build awareness and encourage adoption among different practices.
2011	Krist [86]	Descriptive				1	1	Developed an interactive preventive health record (IPHR) designed to more deeply engage patients in preventive care and health promotion. Within 6 months, practices had encouraged 14.4% of patients to use the patient portal (ranging from 1.5% to 28.3% across 14 practices). Practices incorporated the patient portal into workflow and used it to prepare patients for visits, explain test results, automatically issue patient reminders for overdue services, prompt clinicians about needed services, and formulate personalized prevention plans.
2011	Levy [35]	Descriptive	1					As of December 2010, in the KP Northern California Region, 64% of all eligible health plan members (n=3.6 million) had signed up for online access. Users are 60% female and 40% male. The median age is 48 but ranges from 13 to 95. Secure messaging was associated with a decrease in office visits, an increase in measurable quality outcomes (in primary care), and excellent patient satisfaction.
2011	McInnes [34]	Qualitative	1	1		1		Patients believed that electronic information would be more convenient and understandable than information provided verbally. Patients saw little difference between messages about HIV versus about diabetes and cholesterol. Providers expected increased workload from the electronic outreach and wanted additional primary care resources and methods to support the flow of patients screening sign-up.
2011	North [33]	Cohort study	1					Of the 12,050 office appointments examined, portal registrations within 45 days of the appointment were 11.7% for instructional video, 7.1% for paper instructions, and 2.5% for control respectively. Within 6 months following the interventions, 3.5% in the video cohort, 1.2% in the paper, and 0.75% of the control patients demonstrated portal use by initiating portal messages to their providers.
2011	Sarkar [32]	Descriptive	1					After adjusting for age, gender, race/ethnicity, immigration status, educational attainment, and employment status, compared to non-Hispanic Caucasians, African Americans and Latinos had higher odds of never logging on (OR 2.6 (2.3 - 2.9); OR 2.3 (1.9 - 2.6)), as did those without an educational degree (OR compared to college graduates, 2.3 (1.9 - 2.7)).
2011	Sequist [30]	RCT	1			1	1	Of 552 patients randomized to receive the intervention, 296 (54%) viewed the message, and 47 (9%) used the Web-based risk assessment tool. Among those who viewed the electronic message, risk tool users were more likely than nonusers to request screening exams and to be screened. Screening rates were higher at 1 month for patients who received electronic messages than for those who did not, but this difference was no longer significant at 4 months.

2011	Shaw [112]	Descriptive				1		Data from this study suggest that a significant portion of patients (29.7%) with diabetes utilize the portal (n=2190). Clinical outcome results indicated that portal use was not a significant predictor of low-density lipoprotein and total cholesterol levels. However, portal use was a statistically significant predictor of glycosylated hemoglobin (HbA1c).
2011	Wynia [76]	Descriptive		1				Results from a national survey of physicians in 2008-09 found 64% had never used a patient portal, 42 % interested in trying it (n=865). Rural physicians expressed much more willingness to use such records compared to urban or suburban physicians. Physicians have concerns about patients' privacy, the accuracy of underlying data, their potential liability for tracking all of the information, and the lack of payment to clinicians for using or reviewing these patient records.
2011	Yau [78]	Qualitative		1				Family practice physicians concerns focused on 3 main themes: data management, practice management, and the patient-physician relationship. Subthemes included security, privacy, reliability of data, workload, physician obligations, patient misinterpretation of medical information, and electronic communication displacing face-to-face visits.
2011	Zulman [31]	Descriptive	1					Of the 18,471 users of My HealtheVet who were survey respondents, 79% were interested in sharing access to their patient portal with someone outside of their health system (62% with a spouse or partner, 23% with a child, 15% with another family member, and 25% with a non-VA health care provider). Among those who selected a family member other than a spouse or partner, 47% lived apart from the specified person.
2012	Delbanco [116]	Quasi-experimental				1		Participants reported that having access to clinical notes helped them feel more in control of their care and helped increase medication adherence; few had privacy concerns or reported that the notes caused confusion, worry, or offense (n=11,797). Post intervention, less than 5% of doctors reported longer visits, with practice size having little effect; 3% - 36% of doctors reported changing documentation content; and 0% - 21% reported taking more time writing notes (n=105).
2012	Emani [47]	Descriptive	1		1	1		Computer use among non-adopters was lower than that among portal users and rejecters. Factors identified by the diffusion of innovation model emerged in the factor analysis: ease of use, relative advantage, observe-ability, and trial-ability. Significant positive predictors of the value of the portal for communicating with the doctor's office: relative advantage, ease of use, trial-ability, perceptions of privacy and security, age, and computer use.
2012	Johnson [40]	Descriptive	1				1	Portal users most often preferred immediate access to reports (n=53): 60.2% for the nearly normal scenario, 47.2% for the seriously abnormal scenario, and 45.3% for the indeterminate scenario. Three-day delayed access was next most commonly preferred: 28.3% and 35.8%, respectively. 79.2% preferred the portal method of notification to phone call or letter. Most would use a variety of educational resources and found alternative lay-language conclusions and hyperlinks helpful.
2012	Krist [90]	RCT				1	1	This randomized controlled trial involved 8 primary care practices. At 4 and 16 months, 10.2% and 16.8% of invited patients used the patient portal (n=4500). At 16 months, 25.1% of users were up-to-date with all services, double the rate among nonusers. At 4 months, delivery of colorectal, breast, and cervical cancer screening increased by 19%, 15%, and 13%, respectively, among users.
2012	Nagykaldi [37]	RCT	1			1		Both patient activation and participants' perception of patient-centeredness of care increased significantly in the portal group compared with control. A greater proportion of portal users received all recommended preventive services; took low-dose aspirin if indicated despite having fewer visits over the study period compared with those in the control group.

2012	Nielsen [38]	Descriptive	1		1			Portal users with Multiple Sclerosis tended to be young professionals with minimal physical disability. The most frequently used portal feature was secure patient-physician messaging. Message content mostly consisted of medication or refill requests and self-reported side effects. Independent predictors and barriers of portal use include the number of medications prescribed by our staff, Caucasian ethnicity, arm and hand disability, and impaired vision.
2012	Noblin [42]	Descriptive	1				1	The key variable explaining patient willingness to adopt a patient portal was the patient's health literacy as measured by the eHealth Literacy Scale (eHEALS). Adoption and use rates may also depend on the availability of office staff for hands-on training as well as assistance with interpretation of medical information.
2012	Palen [115]	Descriptive				1		Having online access to medical records and clinicians was associated with increased use of clinical services: increase in the per-member rates of office visits and telephone encounters, after-hours clinic visits, emergency department encounters and hospitalizations for patient portal users vs. nonusers.
2012	Rosenbloom [41]	Pilot study	1			1		A pilot version of Flu Tool was deployed for a 9-week period in the 2010-2011 influenza season. Flu Tool was accessed 4040 times, and 1017 individual patients seen in the institution were diagnosed as having influenza.
2012	Schnipper [114]	RCT				1		The proportion of medications per patient with unexplained discrepancies was 42% in the portal medication review tool group vs. 51% in the control arm (adjusted OR 0.71, 95% CI 0.54 to 0.94, p=0.01). The number of unexplained discrepancies per patient with potential for severe harm was 0.03 in the intervention arm and 0.08 in the control arm (adjusted RR 0.31, 95% CI 0.10 to 0.92, p=0.04).
2012	Tenforde [39]	Descriptive	1					Compared to non-users, portal users were younger, had higher incomes and education level, Caucasian, and had better unadjusted and adjusted diabetes quality measure profiles (n=10,746). Increasing number of login days was generally not associated with more favorable diabetes quality measure profiles among portal users.
2012	Tsai [46]	Descriptive	1					71% of veterans reported using the internet and nearly a fifth reported using HealtheVet (n=7215). Veterans who were younger, more educated, white, married, and had higher incomes were more likely to use the internet. There was no association between background characteristics and use of HealtheVet. Mental health service users were no less likely to use the internet or HealtheVet than other veterans.
2012	Urowitz [45]	Qualitative	1	1	1			Online chronic disease management portals increase patient access to information; improvements in the portal itself may improve usability and reduce attrition. This study identifies a grey area that exists in the roles that providers should play in online disease management. Identified themes: facilitators of disease management, barriers to portal use, patient-provider communication and relationship, and recommendations for portal improvements.
2012	Wagner [44]	RCT	1			1		No impact of the patient portal was observed on blood pressure, patient activation, patient perceived quality, or medical utilization in the intention-to-treat analysis. Younger age, self-reported computer skills, and more positive provider communication ratings were associated with frequency of portal use.
2012	Wakefield [43]	Descriptive	1			1		Those who enroll and use a patient portal have different demographic characteristics and interests levels in selected portal functions (e.g., e-mailing providers, viewing medical records online, making appointments) and initially perceive only limited improvements in care because of the portal.
2012	Wright [113]	RCT				1		Intention-to-treat analysis showed that patients in the intervention arm were significantly more likely to receive mammography (48.6% vs. 29.5%) and influenza vaccinations (22.0% vs. 14.0%)(n=3,979). No significant improvement was observed in rates of other screenings.

2013	Ahlers-Schmidt [48]	Descriptive	1		1			Following demonstration, the 97% of parents surveyed thought the patient portal was easy to use and planned to view medical records and laboratory results (n=171). They disliked having separate accounts for each child and the lack of a ''symptom checker'' and wanted access to the patient portal via on-site kiosks.
2013	Britto [124]	Qualitative				1		Consistent access to their child's medical records provided parents with a sense of control and security and reduced anxiety. Access to lab results enabled parents to be proactive when making treatment decisions, which also helped alleviate anxiety. Some parents felt that information obtained through the portal provided reassurance that they were doing well managing their child's illness and making progress.
2013	Butler [58]	Descriptive	1					Rogers' Diffusion of Innovations theory was used to frame perceptions of portal use by clinic staff and likely users. Researchers and care teams promoting adoption among clinicians should consider implementing systems compatible with existing systems. When this isn't possible, teams should demonstrate trial-ability by creating specific, easy procedures for communicating and tracking modification requests. Some participants may have been agents for change in the positive, but others were negative about the system. Teams should use "the tendency for groups to self-organize around agents of change" to encourage patient portal champions within the clinic.
2013	Gu [59]	Qualitative	1			1		Patients used their portal if they could interact with their provider (e.g. order a repeat prescription, review laboratory results, medication list). Perceived usefulness of the portal was strong, especially if linked to a self-care action. Intent-to-use was not adversely affected by difficulties in using some aspects of the tool, if the perceived usefulness was strong.
2013	Hanberger [57]	RCT	1			1		51% of participants visited the pediatric diabetes specific portal the first study year and 35% the second study year (n=169). Patients, who had someone in the family visiting the portal 5 times or more, had shorter diabetes duration, were younger, had lower HbA1c after 1 year of access, and were more often girls. Peer interaction was identified as valued aspect.
2013	Heyworth [123]	Pilot study				1		Nearly 50% of the potential adverse drug events were classified as serious (n=23). Overall, participants were enthusiastic about medication reconciliation tool; 90% said they would use it again (n=60). Enabling patients to conduct medication reconciliation through a web portal is feasible in the transition from inpatient to outpatient care and may improve medication safety.
2013	Kerns [56]	Qualitative	1			1		For themes identified (relevance, trust and functionality) participants indicated that endorsement and use of the portal by their personal clinician was vital. In particular, participants' comments linked the portal use to: (1) integrating the portal into current care, (2) promoting effective patient-clinician encounters and communication and (3) their confidence in the accuracy, security and privacy of the information.
2013	Ketterer [55]	Descriptive	1					Odds of portal activation was decreased for: Medicaid and uninsured vs. private insurance, black and other vs. white race, Hispanic ethnicity, and increased for: infant age vs. school age, living more than 8.8 miles away from the practice, having more office encounters, and having 3 or more items on the problem list.
2013	Lyles [54]	Descriptive	1					Those reporting greater trust were more likely to be registered portal users or SM users. Better communication ratings were also related to being a registered user among older patients.
2013	Monkman [88]	Descriptive			1		1	Heuristic evaluation and post hoc analysis of a users interacting with a patient portal system revealed health literacy issues and demonstrated ability to use such methods to understand health literacy implications in design of portal systems.
2013	Murray [122]	Pilot study				1		New documentation of family health history entered via patient portal was significantly higher than the control group after adjustment for differences in socio-demographic characteristics.

2013	Nazi [77]	Qualitative		1				Healthcare professionals identified SM as the missing element of complex information landscape and its implementation acted as a catalyst for change. SM was found to have important consequences for access, communication, patient self-report, and patient/provider relationships.
2013	Nazi [53]	Pilot study	1			1		84% of veteran participants enrolled in the pilot program believed the information and services were helpful (n=688). The most highly ranked feature was access to personal health information from the VA EHR. 72% of respondents indicated that the pilot portal made it easy for them to locate relevant information. 66% agreed that the pilot program helped improve their care, with 90% indicating that they would recommend it to another Veteran.
2013	North [120]	Descriptive				1		Patients use portal messages 3.5% of the time for potentially high-risk symptoms of chest pain, breathing concerns, abdominal pain, palpitations, lightheadedness, and vomiting. Death, hospitalization, or an ED visit was an infrequent outcome following a secure message or eVisit. Screening the message subject line for high-risk symptoms was not successful in identifying high-risk message content.
2013	North [121]	Descriptive				1		No significant change in face-to-face visit frequency was observed following implementation of portal messaging. Subgroup analysis also showed no significant change in visit frequency for patients with higher message utilization or for those who had used the messaging feature longer.
2013	Osborn [52]	Mix-method	1			1		Users were more likely than nonusers to be Caucasian/white, have higher incomes, and be privately insured. Users reported using the portal to request prescription refills/reauthorizations and to view their medication list, and they were enthusiastic about the idea of added refill reminder functionality. They were also interested in added functionality that could streamline the refill/reauthorization process, alert providers to fill/refill non-adherence, and provide information about medication side effects and interactions.
2013	Otsuka [119]	RCT				1		Intervention recipients received information about herpes zoster vaccination through mail or through patient portal had significantly higher vaccination rates than controls in both patient portal user and non-patient portal groups.
2013	Pai [82]	Descriptive			1	1		The two most commonly accessed medical records were lab results and provider notes. Of survey respondents, 94% were satisfied with the access to their medical records, 56% said that provider helped to answer their questions, 56% felt that it made communication better with their physicians, 38% found new and useful information, and 88% said that they would continue to use the portal (n=17).
2013	Ronda [51]	Descriptive	1					Among 128 patients with type 1 diabetes, those with a login (89.8%) were younger and more frequently treated by an internist. In 1,262 patients with type 2 diabetes, fewer patients had a log-in (41.0%). The likelihood of having a login was independently associated with younger age, male gender, higher educational level, and treatment by an internist, longer duration of diabetes, and poly-pharmacy.
2013	Sanders [50]	Descriptive	1					Only 12% respondents (n=654) within safety-net practices had no experience using a computer. Age, sex, and race were not associated with interest in a portal. A majority of patients have access to the Internet and are interested in using a portal to manage their care, but they are not prepared.
2013	Sarkar [118]	Descriptive				1		Non-adherence among statin users decreased only among patients initiating occasional or exclusive use of the refill function. In adjusted models, non-adherence declined by an absolute 6% among exclusive users, without significant changes among occasional users (n=8,705). Similar LDL decreases were also seen among exclusive users.

2013	Schickedanz [49]	Descriptive	1					60% of patients used email, 71% were interested in using electronic communication with health care providers, and 19 % reported currently using email informally with these providers for health care (n=408). Those already using any email were more likely to express interest in using it for health matters. Most patients agreed electronic communication would improve clinic efficiency and overall communication with clinicians.
2013	Taha	Descriptive Usability testing			1			Participants in both age groups (middle age and older adults) experienced significant difficulties in using the portal to complete routine health management tasks. Older adults, particularly those with lower numeracy and technology experience, encountered greater problems using the system. Cognitive abilities predicting task performance varied according to the complexity of the task.
2013	VanDerVaart	Mix-method		1		1		Interviewed multidisciplinary team identified potential advantages and challenges to patient portal use. Most respondents reported that data on diagnosis, medication, and treatment plan and consultations should be released to patients, but opinions differed considerably about more complex data, clinical notes, lab results and radiological images.
2013	WadeVuturo [117]	Mix-method				1		Self-reported benefits of SM within a portal included enhanced patient satisfaction, efficiency and quality of face-to-face visits, and access to clinical care outside traditional face-to-face visits. Self-reported barriers to using SM within a portal included preconceived beliefs or rules about SM and prior negative experiences with SM. Participants' assumptions about providers' opinions about SM and providers' instructions about SM also influenced use.
2013	Woods [87]	Qualitative				1	1	Patients felt that seeing their full medical records positively affected communication with providers and the health system enhanced knowledge of their health and improved self-care.
2013	Zarcadoolas [89]	Qualitative			1	1	1	Consumers were most positive about features that increased convenience, such as making appointments and refilling prescriptions. Consumers raised concerns about a number of potential barriers to usage, such as complex language, complex visual layouts, and poor usability features.
2014	Abramson [67]	Descriptive	1					74% of survey respondents reported that they would use a patient portal and wanted a broad array of functionalities available (n=701). Potential portal use was significantly associated with Internet use at least monthly, a belief that portals may improve the security of health information, and a belief that they may improve quality of care.
2014	Aberger [125]	Case report				1		Description of a telehealth system that incorporates home electronic blood pressure monitoring that uploads to a patient portal coupled with a Web-based dashboard that enables clinical pharmacist collaborative care in a renal transplant clinic. Preliminary results show statistically significant reductions in average systolic and diastolic BP of 6.0 mm Hg and 3.0 mm Hg, respectively, at 30 days after enrollment.
2014	Ancker [71]	Descriptive	1					Portal users were no different from nonusers in patient activation. Portal users did have higher education level and more frequent Internet use, and were more likely to have precisely 2 prescription medications (verse one or more).
2014	Ancker [66]	Descriptive						Survey of 800 respondents in consecutive years (2012-13) of the Empire State Poll, an annual random-digit-dial telephone survey. Reported rate of portal use rose from 11% in 2012 to 17% in 2013. The proportion of portals provided by doctors and healthcare organizations increased sharply (from 50% in 2012 to 73%); there was decrease in the proportion provided by insurers.
2014	Barron [126]	Descriptive-Usability			1	1		Older adults with chronic obstructive pulmonary disease or congestive heart failure were evaluated along with their caregivers. Cognitive walkthrough of 14 patients and 19 caregivers were evaluated. Patients required more time on all tasks than caregivers. Patient requested written instructions for using the system and a guide for interpreting lab results. Caregiver suggestions included improving color contrast and presentation of the current medication list.

2014	Borbolla [127]	Descriptive				1		Use of the Infobutton Standard to integrate MedlinePlus information in institutional patient portal. One year of usage data demonstrated that patients accessed MedlinePlus information in Spanish at a similar rate to other personalized information generated locally. Infobuttons associated to laboratory test results were used in approximately 10% of patients' portal sessions when reviewing lab results. The lab test that triggered most of the Infobutton uses was complete blood count, followed by urine culture.
2014	Cabitza [137]	Descriptive				1		Most popular features for providers are information sharing with other providers, pushing reminders to patients, sharing clinical notes and labs, and secure messaging. For patients, making appointments, receiving reminders, the ability to enter personal information and secure messaging were the most popular features.
2014	Chrischilles [136]	RCT				1		Compared with low/non-users, high users reported significantly more changes in medication use and improved medication reconciliation behaviors, and recognized significantly more side effects. There was no difference in use of inappropriate medications or adherence measures.
2014	Crotty [135]	Descriptive				1		At the end of 2010, 22.7% of all patients seen within a large academic health system had enrolled in the portal, and 36.9 % of enrolled patients (8.4% of all patients) had sent at least one message to a physician (n=49,778). Physicians saw a near tripling of e-mail messages during the study period; the number of messages per hundred patients per month stabilized between 2005 and 2010 (average of 18.9 messages).
2014	Druss [134]	RCT				1		170 patients with a serious mental disorder and a comorbid medical condition treated in a community mental health center were randomly assigned to either a patient portal or usual care. In the patient portal group, the total proportion of eligible preventive services received increased from 24% at baseline to 40% at the 12-month follow-up, whereas it declined in the usual care group, from 25% to 18% (n=85). The significant differences in the quality of medical care may be explained by the increase in the number of outpatient medical visits among portal users.
2014	Hall [133]	Descriptive (feasibility study)	1					Patients hospitalized for resection of colorectal or endometrial cancer was recruited to receive their mismatch repair result via the patient portal. 81% of participants who had a result posted to their electronic patient health record completed follow-up, surpassing feasibility thresholds (n=36). Ineligibility was more common among non-white patients and patients ≥65 of age due to either low Internet use or access to the Internet.
2014	Gerber [60]	Descriptive	1			1		6,495 patients enrolled in MyChart (National Cancer Institute patient portal) from 2007 to 2012. There were a total of 5,942,501 patient MyChart actions. The most common portal actions were viewing test results (37%), viewing and responding to clinic messages (29%), and sending medical advice requests (6.4%). Increased portal use was significantly associated with younger age, white race, and an upper aero-digestive malignancy diagnosis. 37% of all log-ins and 31% of all medical advice requests occurred outside clinic hours. Over the study period, the average number of patient log-ins per year more than doubled.
2014	Robotham [98]	Descriptive			1	1		Description the development of an ePHR for people with severe mental health problems. Portal users and clinicians preferred interactive features such as access to care plans and care notes, a mood tracker, patient reported outcomes feeding into the clinical record, and social networking features. Users positively rated the usability of the portal.
2014	Haun [64]	Mix-method	1		1	1	1	Veterans identified and demonstrated barriers to successful portal usage that can be addressed with education, skill building, and system modifications. Analysis of secure message content data provided insights to reasons for use that were not disclosed by participants during interviews, specifically sensitive health topics such as erectile dysfunction and sexually transmitted disease inquiries.

2014	Hess [132]	Descriptive				1		Participants with high cardiovascular disease risk from 73 primary care practices received notifications via a patient portal regarding prevention gaps (i.e., unmet preventive healthcare or chronic disease monitoring). Of the 584 patients, 86% received at least one reminder; 61% of these participants accessed the portal or received the care that triggered the message after the first message and 73% after the first two messages. Of the 2,656 prevention gaps these participants had over 1 year, 1,539 (58%) were closed.
2014	Jung [63]	Descriptive	1					On average, eVisit via patient portal adopters were younger and predominantly female. Patients who are familiar with the patient portal are more likely to use the service, as are patients with more complex health issues.
2014	Krist [62]	Mixed Method	1					A proactive and customized implementation strategy designed by 8 practices resulted in 25.6% of patients using the patient portal, with the rate increasing 1% per month over 31 months (n=112,893). 23.5% of patient portal users signed up within 1 day of their office visit. Older patients and patients with comorbidities were more likely to use the patient portal; blacks and Hispanics were less likely. Older age diminished as a factor after adjusting for comorbidities. Implementation by practice varied considerably based on clinician characteristics and workflow innovations adopted by practices to enhance uptake
2014	Kondylakis [61]	Pilot study	1					Describes the development and validation of a profile mechanism (ALGA-C Profiler) that measures and collects psycho-cognitive information about patients. Techniques for exploiting the constructed patient profile via a patient portal are discussed. Predefined rules will use a patient's profile to personalize the contents of the information presented and to customize users tasks.
2014	Lau [131]	Descriptive				1		The portal provided access to diabetes education material, personal lab values and a messaging system allowing communication with staff. Patients who logged in 1 or more times were defined as portal users. Compared to non-users, a higher proportion of users achieved A1C ≤7% at follow up.
2014	Nazi [130]	Descriptive				1		33% of surveyed VA patient portal user respondents knew that clinical notes could be viewed, and nearly 23.5% said that they had viewed their notes at least once (n=37,103). The majority of users agreed that accessing their notes will help them to do a better job of taking medications as prescribed (80.1%) and be better prepared for clinic visits (88.6%). 91% of users agreed that use of visit notes would better help them understand their conditions and remember the plan for their care. Users who had either contacted their provider or healthcare team (11.9%) or planned to (13.5%) primarily wanted to learn more about a health issue, medication, or test results (53.7%).
2014	Neuner [69]	Descriptive	1			1		Overall portal enrollment in a recently adopting fee-for-service multispecialty system increased from 13.2% to 23.1% between 2010 and 2012, but varied substantially by physician specialty. In 2013, more than 97% of physicians would have met requirements for a stage 2 MU utilization measure, but only 38% of all physicians (87% of primary care physicians and 37% of other specialists) would have met e-mail requirements. Most valued features to patients were appointment scheduling, ability to view problem list and test results, preventative care reminders and secure messaging.
2014	Phelps [68]	Descriptive	1					At the time of analysis, 11,352 patients were registered patient portal users for 0-42 months (median 17). More than half became persistent users; the median login was 2 times per month. Supporting patients through the first steps of establishing access to their online records is associated with higher rates of long-term use of patient portal and likely would increase use of other electronic health records provided for patients with chronic disease.

2014	Riippa [70]	Descriptive	1			1		No significant effect of access to patient portal on patient activation was detected. Results suggest that the intervention had greatest effect on patients starting at the highest level of patient activation, and among patients diagnosed within a year of the intervention.
2014	Taha [73]	Descriptive			1		1	Results indicated that numeracy and Internet experience had a significant impact on older adults' ability to perform portal tasks and that they tended to overestimate their numeracy skills.
2014	Tom [129]	Descriptive	1					Children whose parents used 1 or more portal features had higher odds of adhering to the recommended immunizations only at KP Northwest. Portal use was associated with better adherence to well-child care visit recommendations for both KP Hawaii and KP Northwest.
2014	VanDerVaart [65]	Descriptive	1		1	1	1	Age, amount of Internet use and self-perceived Internet skills significantly predicted portal use. Of the respondents who had logged in, 44% reported feeling more involved in their treatment and 37% felt they had more knowledge about their treatment (n=360). Significant differences over time were not found on the empowerment-related instruments.
2014	Wright [113]	Descriptive				1		Patient attitudes towards the problem list function were positive overall. Nearly half (45.6%) of patients identified at least one major or minor problem missing from their list (n=3,649). After viewing the list, 56.1% of patients reported taking at least one action in response. 67%of patients reported feeling at least somewhat happy while viewing their problem list, though others reported feeling sad (30.4%), worried (35.7%) or scared (23.8%), few patients reported feeling angry (16.6%) or ashamed (14.3%).
